# Supplementary material for: Integrated phylogenomic and fossil evidence of stick and leaf insects (Phasmatodea) reveal a Permian–Triassic co-origination with insectivores
Source: R Soc Open Sci. 2020 Nov 11;7(11):201689. doi: 10.1098/rsos.201689 (PMC7735357; doi:10.1098/rsos.201689)
Supplement: Supplementary Information [file rsos201689supp1.pdf]

Supplementary Information

Phylogenomics of stick and leaf insects (Phasmatodea) reveal a Permian-Triassic co-origination with early insectivores

Erik Tihelka, Chenyang Cai, Mattia Giacomelli, Davide Pisani & Philip C. J. Donoghue

Contents

Phylogenetic and age justification of fossil calibrations 3

Figure S1 | Overview of nodes calibrated with fossils 8

Figure S2 | Stick and leaf insect phylogeny inferred from a LG4X + R re-analysis of the Simon et al. (2019) transcriptome dataset, excluding distantly related outgroups 9

Figure S3 | Stick and leaf insect phylogeny inferred from a CAT-GTR + G re-analysis of the Simon et al. (2019) transcriptome dataset with full outgroup taxon sampling 10

Figure S4 | Dated phylogenetic tree of Phasmatodea based on the PhyloBayes re-analysis of the transcriptome dataset of Simon et al. (2019), excluding distantly related outgroups and using five fossil calibrations, i.e. without *Echinosomiscus primoticus.* Uniform prior distribution, independent rate clock. 11

Figure S5 | Dated phylogenetic tree of Phasmatodea based on the PhyloBayes re-analysis of the transcriptome dataset of Simon et al. (2019), excluding distantly related outgroups and using six fossil calibrations, i.e. with *Echinosomiscus primoticus.* Uniform prior distribution, autocorrelated rate clock. 12

Figure S6 | Dated phylogenetic tree of Phasmatodea based on the PhyloBayes re-analysis of the transcriptome dataset of Simon et al. (2019), excluding distantly related outgroups and using five fossil calibrations, i.e. without *Echinosomiscus primoticus.* Uniform prior distribution, autocorrelated rate clock. 13

Figure S7 | Dated phylogenetic tree of Phasmatodea based on the PhyloBayes re-analysis of the transcriptome dataset of Simon et al. (2019), excluding distantly related outgroups and using six fossil calibrations. Uniform prior distribution, independent rate clocks. Cauchy 10%, autocorrelated rate clock. 14

Figure S8 | Dated phylogenetic tree of Phasmatodea based on the PhyloBayes re-analysis of the transcriptome dataset of Simon et al. (2019), excluding distantly related outgroups and using six fossil calibrations. Uniform prior distribution, independent rate clocks. Cauchy 10%, independent rate clock. 15

Figure S9 | Dated phylogenetic tree of Phasmatodea based on the PhyloBayes re-analysis of the transcriptome dataset of Simon et al. (2019), excluding distantly related outgroups and using six fossil calibrations. Uniform prior distribution, independent rate clocks. Cauchy 50%, autocorrelated rate clock. 16

Figure S10 | Dated phylogenetic tree of Phasmatodea based on the PhyloBayes re-analysis of the transcriptome dataset of Simon et al. (2019), excluding distantly related outgroups and using six fossil calibrations. Uniform prior distribution, independent rate clocks. Cauchy 50%, independent rate clock. 17

Figure S11 | Dated phylogenetic tree of Phasmatodea based on the PhyloBayes re-analysis of the transcriptome dataset of Simon et al. (2019), excluding distantly related outgroups and using six fossil calibrations. Uniform prior distribution, independent rate clocks. Cauchy 90%, autocorrelated rate clock. 18

Figure S12 | Dated phylogenetic tree of Phasmatodea based on the PhyloBayes re-analysis of the transcriptome dataset of Simon et al. (2019), excluding distantly related outgroups and using six fossil calibrations. Uniform prior distribution, independent rate clocks. Cauchy 90%, independent rate clock. 19

Supplementary references 20

Phylogenetic and age justification of fossil calibrations

1.1 Crown Mantophasmatodea (162.5 Ma – 326 Ma), node 1

1.1.1 Fossil taxon and specimen. *Juramantophasma sinica* Huang, Nel, Zompro & Waller, 2008 (Mantophasmatidae) [NIGP 142171a, b (holotype): Nanjing Institute of Geology and Palaeontology, Chinese Academy of Sciences, Nanjing, China]. Locality near the Daohugou Village, Wuhua Township, Ningcheng County, Chifeng City, Middle Jurassic Haifanggou Formation, Inner Mongolia, north-east China (Huang et al. 2008).

1.1.2 Phylogenetic justification. *J. sinica* is known from a dorsoventral compression fossil preserving an adult female. It displays several apomorphies of Mantophasmatidae, namely the third tarsomere with a sclerotized elongated dorsal process; enlarged and fan-like pretarsal arolia with a clearly visible row of dorsal setae; last tarsomere connected to the penultimate one at a right angle; and female gonoplacs short and claw-shaped (Zompro et al. 2002; Huang et al. 2008). The fossil differs from extant Mantophasmatidae by the absence of ventroapical spines on the tibiae, which are characteristic of Mantophasmatinae (Zompro 2005). It can however be placed into the extinct subfamily †Raptophasmatinae known otherwise only from Baltic amber (Zompro 2005, 2008). This makes *Juramantophasma* a member of crown group Mantophasmatodea. The monophyly of Mantophasmatodea is supported by a molecular phylogeny based on 1,300 bp of mitochondrial DNA (Damgaard et al. 2008) and by a total evidence phylogenetic study based on three genes and 125 morphological characters (Terry & Whiting 2005).

1.1.3 Minimum age and justification. The fossil was recovered from the Daohugou beds of the Haifanggou Formation, Inner Mongolia, northeastern China. The precise age of the Daohugou beds has been controversial (He et al. 2004; Gao and Ren 2006; Wang et al. 2005; Zhang 2015). 39Ar–40Ar and SHRIMP U-Pb dating has provided an age of 165 ± 2.5 Ma of the acid volcanic rock overlaying the Daohugou fossiliferous horizon yielding insects (Chen et al. 2004; Liu et al. 2004). This corresponds to the Callovian of the Middle Jurassic and is further corroborated by the composition of the fossil insect community, which is similar to the slightly younger deposit in Karatau, Kazakhstan (Zhang 2015). This provides a minimum age of 162.5 Ma.

1.1.4 Soft maximum age and justification. Given the exceptional rarity of mantophasmatodean fossils, both as compressions and as amber inclusions, it is difficult to set a maximum age constrain on the node. No stem Mantophasmatodea fossils are known to date. On the other hand, the sister group of Mantophasmatodea, the Grylloblattodea (Misof et al. 2014), has a much more extensive fossil record going back to the Late Carboniferous (Cui et al. 2019). The earliest uncontroversial stem grylloblattid is *Sinonamuropteris ningxiaensis* from the Tupo Formation of northwestern China (Peng et al. 2005). Crown Mantophasmatodea and Grylloblattodea is absent from the Tupo Formation and other well-explored late Carboniferous Lagerstätten such as Montceau-les-Mines in France and Mazon Creek in Illinois, USA. We thus consider the maximum age of the Tupo Formation, 326 Ma (Zhang et al. 2013), as a suitable soft maximum constrain for the origin of crown Mantophasmatodea.

1.2 Stem Phasmatodea (162.5 Ma – 295.0 Ma), node 2

1.2.1 Fossil taxon and specimen. *Adjacivena rasnitsyni* Shang, Béthoux & Ren, 2011 (†Susumaniidae) [CNU-PHA-NN2009001 (holotype); CNU-PHA-NN2009002 (paratype): Key Lab of Insect Evolution and Environmental Changes, College of Life Sciences, Capital Normal University, Beijing, China]. Locality near the Daohugou Village, Wuhua Township, Ningcheng County, Chifeng City, Middle Jurassic Haifanggou Formation, Inner Mongolia, north-east China (Shang et al. 2011).

1.2.2 Phylogenetic justification. *A. rasnitsyni*, known from compression fossils of a male and a female preserving the wings and genitalia, displays the following apomorphies of Phasmatodea: ovipositor concealed by an operculum (female specimens); tergum 10 bearing a hook (vomer) on the venter (male specimen) (Bradler 1999, 2009; Tilgner 1999). It is placed outside of crown Phasmatodea by the following combination of wing characters: narrow area between MA2 and MP + CuA1 present; MA2 approaching MP + CuA1 a short

distance before the middle of the forewing; MP + CuA1 forked in the forewing; MA1 fused over a moderate distance with RP in the hindwing; MA2 fused with MA1 distal to its divergence from RP + MA1 in the hindwing (Shang et al. 2011). The monophyly of Phasmatodea is strongly supported by transcriptome data (Misof et al. 2014; Simon et al. 2019), ribosomal and H3 sequences (Terry & Whiting 2005), as well as morphological characters (Friedemann et al. 2012; Bradler 2015).

1.2.3 Minimum age and justification. As in 1.1.3.

1.2.4 Soft maximum age and justification. No convincing stick and leaf insects are known from before the Jurassic. The maximum age constraint is based on the age of the Sakmarian–Artinskian locality Obora in Moravia, Czech Republic (Kukalová 1969). Together with Elmo in Kansas and Tshekarda in Russia, these three localities represent the best-explored and most productive insect Lagerstätten of the Permian (Kukalová-Peck & Tihelka 2019). The lower boundary of the Sakmarian is 295.0 Ma (Ogg et al. 2016).

1.2.5 Discussion. The early evolution of Phasmatodea lies in murky waters; although a considerable number of putative stem phasmatodeans have been reported from the Permian and Mesozoic by workers from the 1960s until the turn of the millennium (Sharov 1968; Carpenter 1992; Rasnitsyn & Quicke 2002; Willmann 2003), mostly on the basis of fragmentary wings. Whether these fossils represent true relatives of modern stick and leaf insects has been questioned (Bradler 2015; Engel et al. 2016). Tilgner (2000) reviewed the fossil record of phasmatodeans and concluded that all the putative stem group fossils from the Palaeozoic and Mesozoic reported at the time were diagnosed based on wing venation characters that are not unique to the order Phasmatodea and are plesiomorphic. It was not until recently that some members of the extinct family †Susumaniidae were recognised as true stem phasmatodeans. Specimens from the Middle Jurassic Jiulongshan (Shang et al. 2011) and Early Cretaceous Yixian Formation in China (Nel & Delfosse 2011; Wang et al. 2014) were described with preserved vomers, male clasping genital structures that represent an apomorphy of Phasmatodea (Bradler 1999, 2009; Tilgner 1999). These fossils can thus be identified as the earlier stem phasmatodeans in the fossil record. Other specimens assigned to †Susumaniidae are known, extending up to the Eocene (Archibald & Bradler 2015), but because the fossil family is not defined by any definitive autapomorphy (Bradler 2015), these fossils should be treated with caution.
Aclistophasma echinulatum, described from the same deposit (Yang et al. 2020), is an equally suitable fossil for calibrating the Phasmatodea – Embioptera split.

1.3 Stem Timematidae (98.17 Ma – 241.5 Ma), node 3

1.3.1 Fossil taxon and specimen. *Tumefactipes prolongates* Chen, Deng, Zhang, Zhang, Ren, Zhu and Gao, 2019 (Timematidae) [BU-001232 (holotype); CNU-PHA-MA2017001 (paratype): Key Lab of Insect Evolution & Environmental Changes, College of Life Sciences, Capital Normal University, Beijing, China]. Burmese amber, Hukawng Valley, Myitkyina District, Kachin State, northern Myanmar (Chen et al. 2019).

1.3.2 Phylogenetic justification. The species, known from more or less complete female specimens preserved as amber inclusions, can be placed into Timematodea on the basis of having the autapomorphic basal three tarsomeres fused, resulting into pseudotrimeric tarsi (Kristensen 1975; Tilgner et al. 1999). They further possess a large scape twice as long as the pedicel; prothoracic sternal apophyses; abdomen not distinctly elongated; and rather short legs. While the fusion of the metanotum with the first abdominal tergum represents an apomorphy of Euphasmatodea (all extant Phasmatodea except Timematidae; Tilgner et al. 1999; Bradler 2009), the abdominal tergum I is in fact not fused with the metanotum in *T. prolongates.* However, *Tumefactipes* differs from extant timematodeans in having an unusually elongate head and thoracic segments and possesses a suite of plesiomorphic characters such as the elongate prothorax and the structure of arolia (Chen et al. 2019). The species poor Timematodea has been repeatedly recovered as monophyletic and as a sister group to the remaining Phasmatodea based on morphological and molecular analyses (Bradler et al. 2003; Whiting et al. 2003; Wheeler et al. 2004; Simon et al. 2019).

1.3.3 Minimum age and justification. The fossil is preserved in Burmese amber mined in the Hukawng Valley in northern Myanmar. Volcanoclastic matrix from the amber-bearing horizon was dated radiometrically to 98.79 ± 0.62 Ma (Shi et al. 2012), which is in line with the age predicted based on palaeontological evidence

(Grimaldi et al. 2002). However, the zircon date should be taken as a lower limit for the age of the amber (Mao et al. 2018) since the method made no use of chemical abrasion which typically results into younger ages (Kryza et al. 2012; Metcalfe et al. 2015). A reliable upper limit on the age of Burmese amber is provided by a juvenile *Puzosia* ammonite trapped in the amber, which indicates that the deposit is at most late Albian in age (Yu et al. 2019). Here we use 98.17 Ma as the conservative minimum age of Burmese amber.

1.3.4 Soft maximum age and justification. We use the maximum age of the Ladinian-Carnian Madygen Formation in Kyrgyzstan, the world's richest Triassic insect Konservat-Lagerstätte (Shcherbakov 2008), as the maximum age constrain on the node (Kohli et al. 2016). Despite the abundance of polyneopteran insects in the deposit, no unambiguous stem phasmatodeans have been reported from the locality (Shcherbakov 2008).

1.4 Crown Diapheromerini (Diapheromeridae) (15.97 Ma – 130.8 Ma), node 4

1.4.1 Fossil taxon and specimen. *Clonistria* sp. (Diapheromeridae) [#O-2-13A (figured specimen): Poinar amber collection, Department of Integrative Biology, Oregon State University, Corvallis, Oregon, USA]. Dominican amber, La Buscar mine, Cenozoic El Mamey Formation, Cordillera Septentrional, Dominican Republic (Poinar 2011).

1.4.2 Phylogenetic justification. A single egg preserved in an amber piece can be confidently assigned to Phasmatodea, since it possesses a distinct operculum and micropylar plate, features that are apparently unique to the order (Clark-Sellick 1997). The egg can be placed into the extant genus *Clonistria* based on its vesicular and open capitulum broadly covering the operculum, which are not present in other Diapheromerinae (Clark-Sellick 1997; Poinar 2011; Bradler et al. 2015). Moreover, a well-preserved nymph assigned to the same genus has also been described from the same deposit (Poinar 2011). Together, these Dominican amber inclusions represent the earliest representatives of Diapheromerinae, which has been recovered as monophyletic based on transcriptome data (Simon et al. 2019), and so they are used to calibrate the divergence between Diapheromerinae and Agathemeridae + Pseudophasmatinae.

1.4.3 Minimum age and justification. Microfossils from the amber-bearing unit have yielded a range of ages, the youngest belonging to the Burdigalian (Iturralde-Vinent & MacPhee 1996, 2019). The amber is very probably contemporaneous with the surrounding rock matrix, since it is found in lignite beds and plant-rich sediments (Iturralde-Vinent & MacPhee 2019), which probably represent the original palaeoenvironment. No older amber-bearing rocks in the region are known and the insect fauna contains a high share of extant genera. The age is moreover supported by the vertebrate fossils in the amber (Sherratt et al. 2015). The minimum age for Dominican amber is thus provided by the upper boundary of the Burdigalian, 15.97 Ma (Ogg et al. 2016).

1.4.4 Soft maximum age and justification. The maximum constraint on the clade is provided by the maximum age of Lebanese amber, that has been dated as late Barremian to early Aptian (Maksoud et al. 2017). This is because none of the Cenozoic and Early Cretaceous Lagerstätten (e.g. Baltic amber, Messel pit, Canadian amber, Taimyr amber, New Jersey amber, Burmese amber, French amber, Spanish amber, Crato Formation, Yixian Formation) preserve any members of the group.

1.5 Crown Anisomorphini (Pseudophasmatidae) (43.47 Ma – 130.8 Ma), node 5

1.5.1 Fossil taxon and specimen. *Eophasmodes oregonense* Clark-Sellick, 1994 [UF 15768-6437 (holotype); UF 15768-6364; 15768-6366; 225-8686 (paratypes): Florida Museum of Natural History, University of Florida, Gainesville, Florida, USA] John Day National Fossil Monument Nut Beds, Eocene Clarno Formation, Oregon, USA (Clark-Sellick 1994).

1.5.2 Phylogenetic justification. The eggs possess a distinct operculum and micropylar plate, features that are apparently unique to the eggs of Phasmatodea (Clark-Sellick 1997). The operculum is tilted ventrally, a

character which is known only in Anisomorphini (Clark-Sellick 1994). The fossil is thus used to calibrate the Anisomorphini – Pseudophasmatini split recovered by analyses of transcriptome data (Simon et al. 2019).

1.5.3 Minimum age and justification. Radiometric dating has yielded a range of ages for the Clarno Nut Bed ranging from 43.76 ± 0.29 Ma to 48.32 ± 0.11 Ma (Swisher 1992; Manchester 1994; Bestland et al. 1999) and this age is further corroborated by the mammal fauna known from the locality (Mihlbachler et al. 2016). This provides a minimum age for the node, 43.47 Ma.

1.5.4 Soft maximum age and justification. As in 1.4.4.

1.5.5 Discussion. The genus was initially described as *Eophasma*, but was later changed to *Eophasmodes* due to the pre-occupation of the original name by a fossil nematode (Clark-Sellick 1997). The genus *Eophasmina*, described from the same locality on the basis of eggs, is an equally suitable candidate for calibration. The fossils do not represent recent contaminants, as all organic content has been completely replaced by silica. Younger Pseudophasmatinae eggs are known from mid-Miocene Dominican amber (Poinar 2011).

1.6 Crown Lonchodidae (98.17 Ma – 167.5 Ma), node 6

1.6.1 Fossil taxon and specimen. *Echinosomiscus primoticus* Engel and Wang, 2016 (family *incertae sedis*) [NIGP 163536 (holotype); CNU-PHA-MA2017001 (paratype): Nanjing Institute of Geology and Palaeontology, Chinese Academy of Sciences, Nanjing, China]. Burmese amber, Hukawng Valley, Myitkyina District, Kachin State, northern Myanmar (Engel et al. 2016).

1.6.2 Phylogenetic justification. *E. primoticus*, known from a male specimen preserved as an amber inclusion, can be placed into Euphasmatodea on the basis of the following apomorphies: having the abdominal sternum I fused with the metasternum; thorn field present on the abdominal tergum 10; and 5-segmented tarsi (Kristensen 1975; Bradler 2009). It can be placed among the 'areolate lineages' on the basis of lacking the area apicalis on the tibiae. It shares with extant Lonchodinae the slender body form and long antennae (Brock & Hasenpusch 2009). Crucially, the tenth abdominal tergite is divided into moveable hemitergites, a character that has traditionally been considered as the hallmark of Lonchodinae (Günther 1953; Beier 1957, 1968; Bradley & Galil 1977; Kevan 1982), although it is absent in the ambiguous taxon *Neohirasea* (Bradler 2009). Extant Lonchodinae are restricted to south east Asia and Australia, which is notable since most of the fauna and flora of Burmese amber is apparently of an Australian origin (Poinar 2019). Admittedly, a similar morphology of the 10th abdominal tergum is also found in Clitumninae (Bradler 2009), but the fossil differs from this subfamily in having the fore femora trapezoid in cross-section, not triangular (Brock & Hasenpusch 2009). Moreover, a morphological phylogenetic analysis recovered *Echinosomiscus* as a sister group to the lonchodin taxon *Eurycantha*, albeit with a rather limited taxon sampling (Yang et al. 2019). *E. primoticus* falls outside of stem Lonchodinae, it differs in the structure of the antennae, head, and abdomen and as such has been placed into its own subfamily †Echinosomiscinae (Engel et al. 2016). We thus provisionally treat the fossil as a sister group to Lonchodinae. The monophyly of Lonchodinae, although controversial in the past, is well-supported by latest transcriptome analyses (Simon et al. 2019).

1.6.3 Minimum age and justification. As in 1.3.3.

1.6.4 Soft maximum age and justification. The maximum constrain on the node is provided by the age of the Daohugou biota in Inner Mongolia, northeastern China (Chen et al. 2004; Liu et al. 2004). No stem phasmatodeans are known from the locality (Huang 2016). Crown phasmatodeans are also absent from the Early Cretaceous Jehol biota in China, Crato Formation in Brazil, Lebanese and Spanish ambers.

1.6.5 Discussion. Other members of Euphasmatodea (Chen et al. 2018) and even supposed euphasmatodean eggs (Rasnitsyn & Ross 2000) have been reported from Burmese amber, although their exact phylogenetic position remains uncertain (Yang et al. 2019). These mid-Mesozoic stick insects together show that Euphasmatodea began to diversify by 100 Ma. The plant seed genus *Knoblochia* known from the Late Cretaceous of central Europe has been recently reinterpreted as possible phasmatodean eggs (Heřmanová et

al. 2013), but this assignment seems unlikely as the supposed eggs lack a distinct micropylar plate (Clark-Sellick 1997) that is characteristic of the order.

Figure S1

Overview of nodes calibrated with fossils. Node 1 – *Juramantophasma sinica*; node 2 – *Adjacivena rasnitsyni*; 3 – *Tumefactipes prolongates*; 4 – *Clonistria* sp.; 5 – *Eophasmodes oregonense*; 6 – *Echinosomiscus primoticus*.

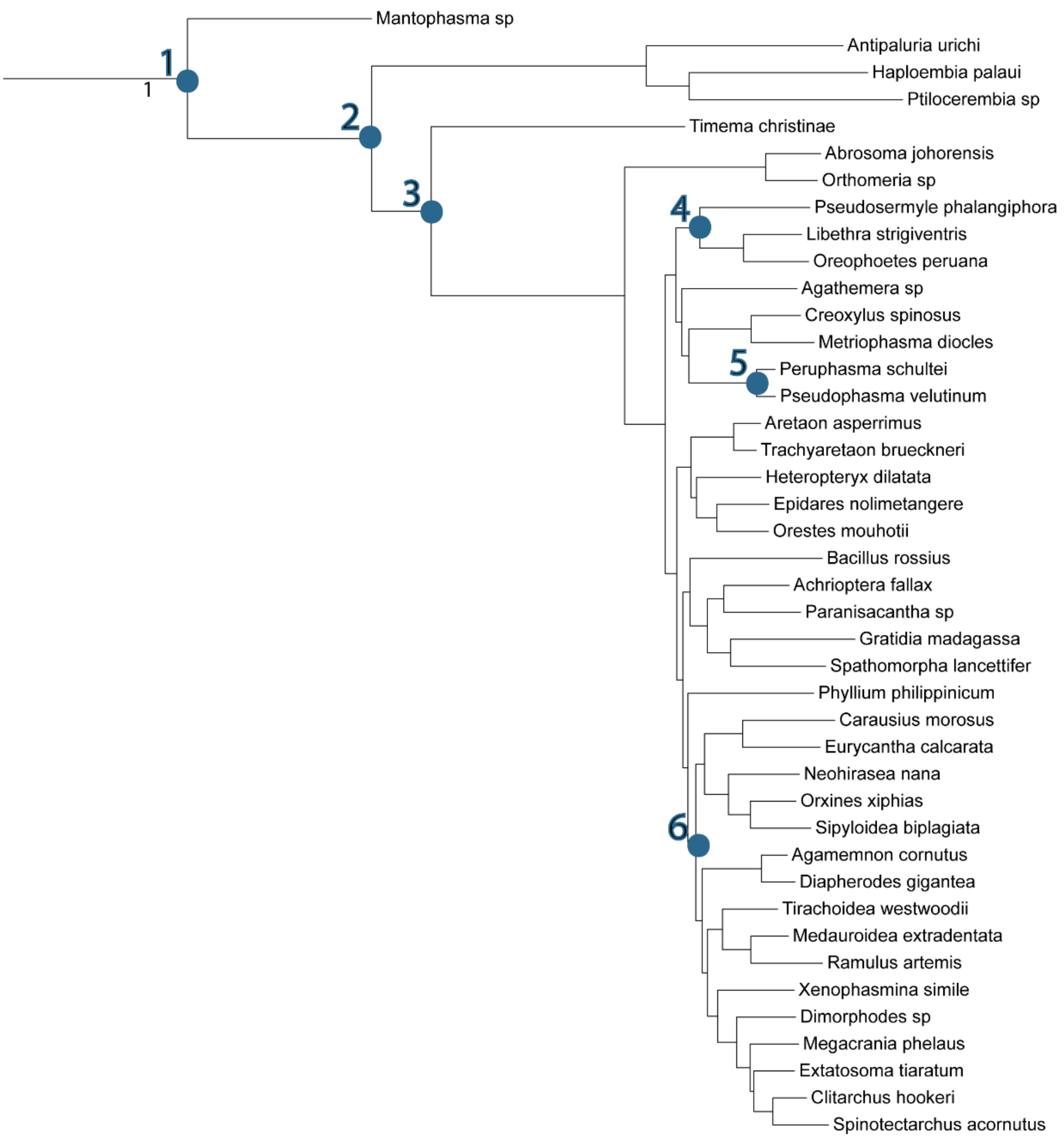

Figure S2

Stick and leaf insect phylogeny inferred from a LG4X + R re-analysis of the Simon et al. (2019) transcriptome dataset, excluding distantly related outgroups.

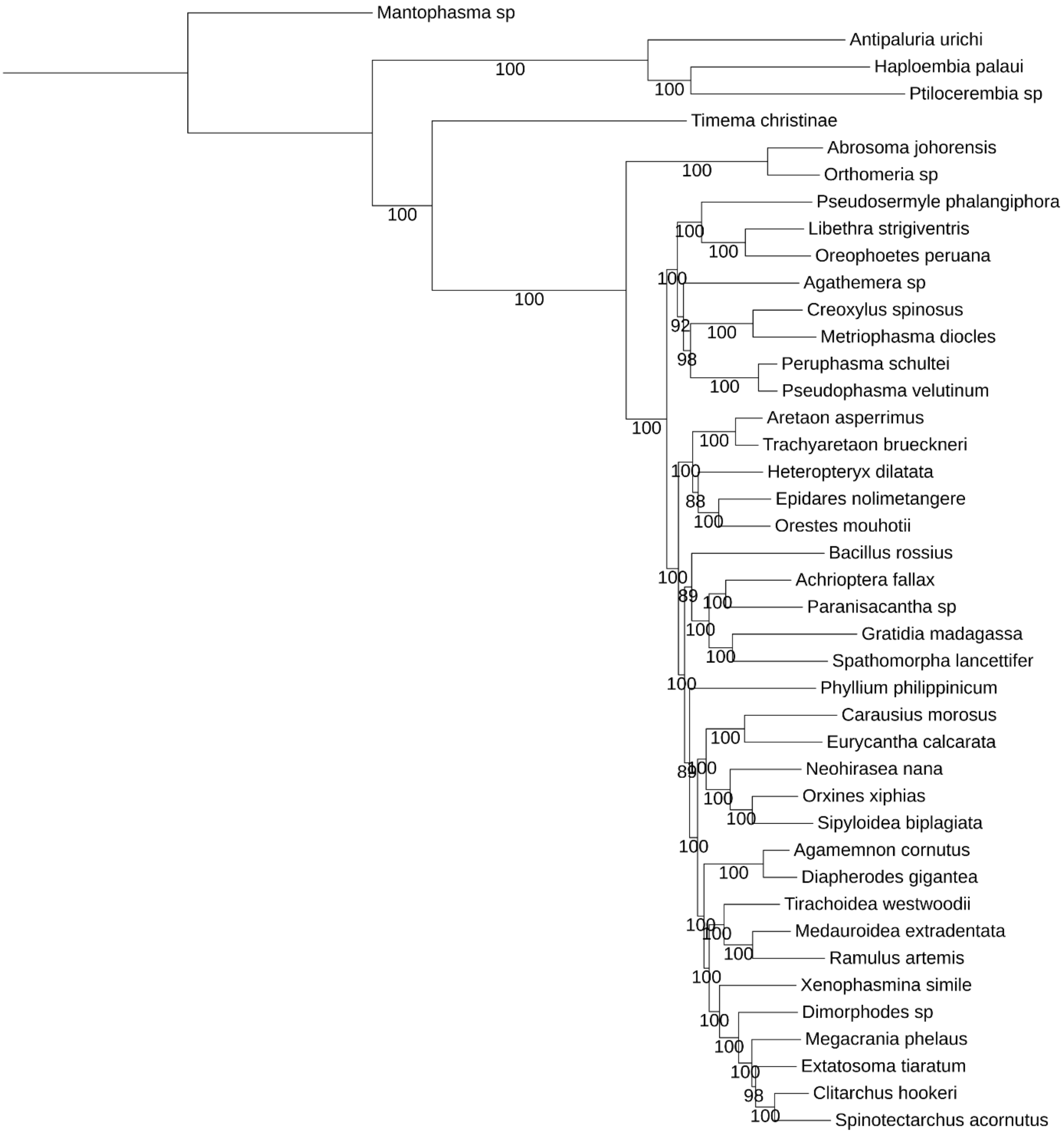

Figure S3

Stick and leaf insect phylogeny inferred from a CAT-GTR + G re-analysis of the Simon et al. (2019) transcriptome dataset with full outgroup taxon sampling

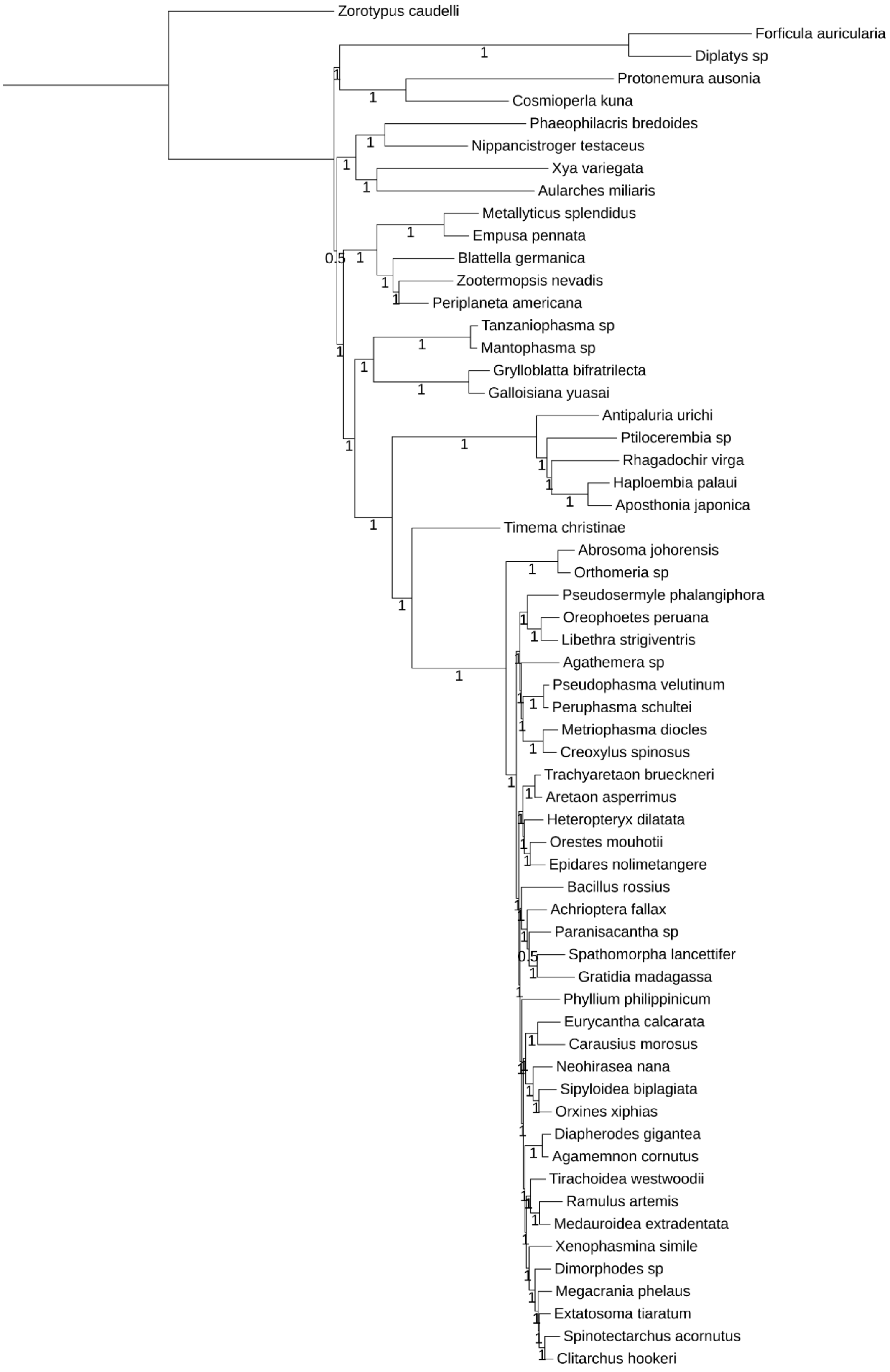

Figure S4

Dated phylogenetic tree of Phasmatodea based on the PhyloBayes re-analysis of the transcriptome dataset of Simon et al. (2019), excluding distantly related outgroups and using five fossil calibrations, i.e. excluding *Echinosomiscus primoticus*. Uniform prior distribution, independent rate clock.

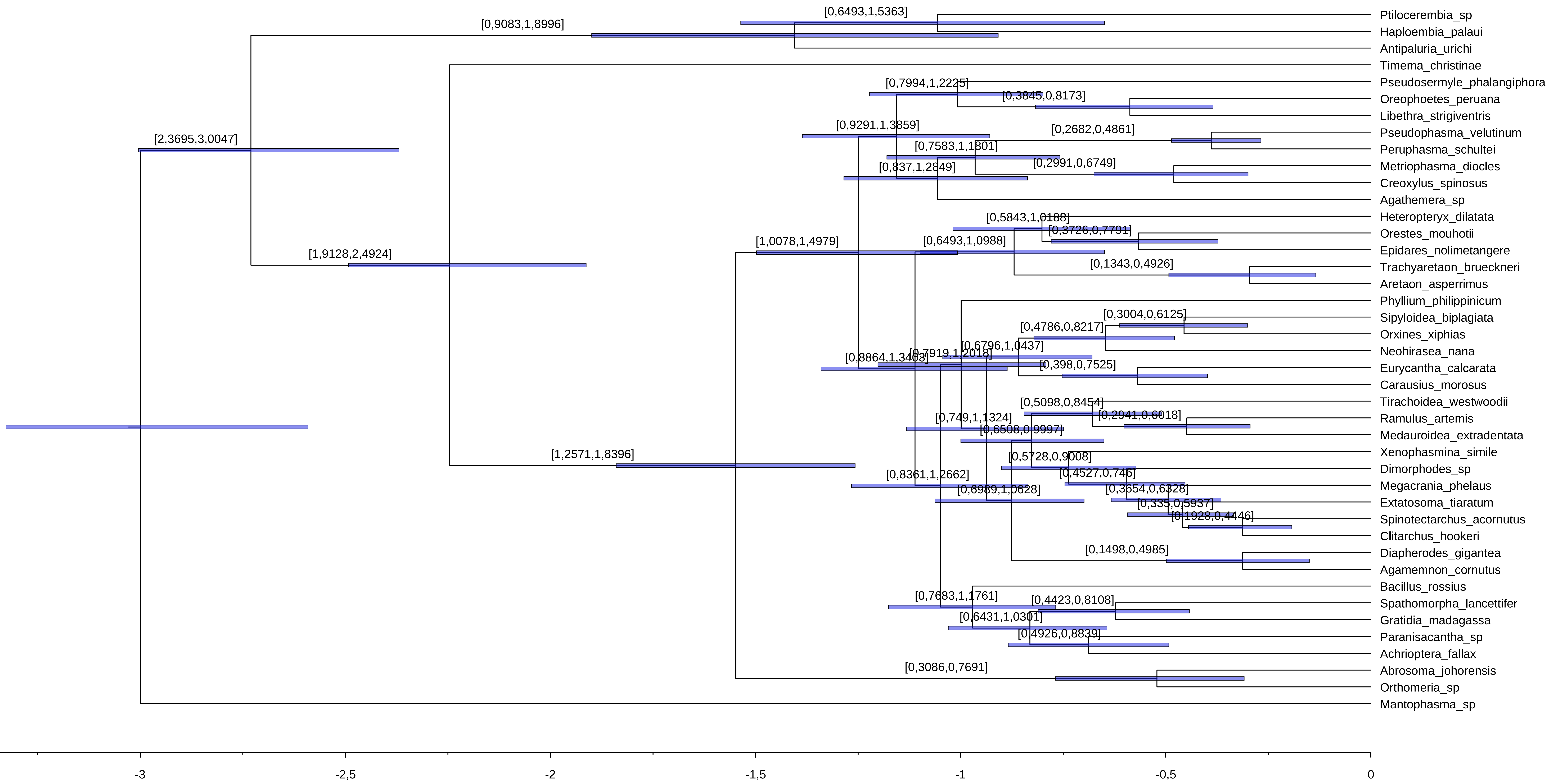
[0,6493,1,5363]
[0,9083,1,8996]
[2,3695,3,0047]
[1,9128,2,4924]
[1,2571,1,8396]
[1,0078,1,4979]
[0,7994,1,2225]
[0,3845,0,8173]
[0,9291,1,3859]
[0,2682,0,4861]
[0,7583,1,1801]
[0,2991,0,6749]
[0,837,1,2849]
[0,5843,1,0188]
[0,3726,0,7791]
[0,6493,1,0988]
[0,1343,0,4926]
[0,3004,0,6125]
[0,4786,0,8217]
[0,6796,1,0437]
[0,7919,1,2018]
[0,8864,1,3403]
[0,398,0,7525]
[0,5098,0,8454]
[0,2941,0,6018]
[0,749,1,1324]
[0,6508,0,9997]
[0,5728,0,9008]
[0,4527,0,746]
[0,8361,1,2662]
[0,6989,1,0628]
[0,3654,0,6328]
[0,335,0,5937]
[0,1928,0,4446]
[0,1498,0,4985]
[0,7683,1,1761]
[0,4423,0,8108]
[0,6431,1,0301]
[0,4926,0,8839]
[0,3086,0,7691]
Ptilocerembia_sp
Haploembia_palaui
Antipaluria_urichi
Timema_christinae
Pseudosermyle_phalangiphora
Oreophoetes_peruana
Libethra_strigiventris
Pseudophasma_velutinum
Peruphasma_schultei
Metriophasma_diocles
Creoxylus_spinosus
Agathemera_sp
Heteropteryx_dilatata
Orestes_mouhotii
Epidares_nolimetangere
Trachyaretaon_brueckneri
Aretaon_asperrimus
Phyllium_philippinicum
Sipyloidea_biplagiata
Orxines_xiphias
Neohirasea_nana
Eurycantha_calcarata
Carausius_morosus
Tirachoidea_westwoodii
Ramulus_artemis
Medauroidea_extradentata
Xenophasmina_simile
Dimorphodes_sp
Megacrania_phelaus
Extatosoma_tiaratum
Spinotectarchus_acornutus
Clitarchus_hookeri
Diapherodes_gigantea
Agamemnon_cornutus
Bacillus_rossius
Spathomorpha_lancettifer
Gratidia_madagassa
Paranisacantha_sp
Achrioptera_fallax
Abrosoma_johorensis
Orthomeria_sp
Mantophasma_sp
-3
-2,5
-2
-1,5
-1
-0,5
0

Figure S5

Dated phylogenetic tree of Phasmatodea based on the PhyloBayes re-analysis of the transcriptome dataset of Simon et al. (2019), excluding distantly related outgroups and using six fossil calibrations, i.e. with *Echinosomiscus primoticus*. Uniform prior distribution, autocorrelated rate clock.

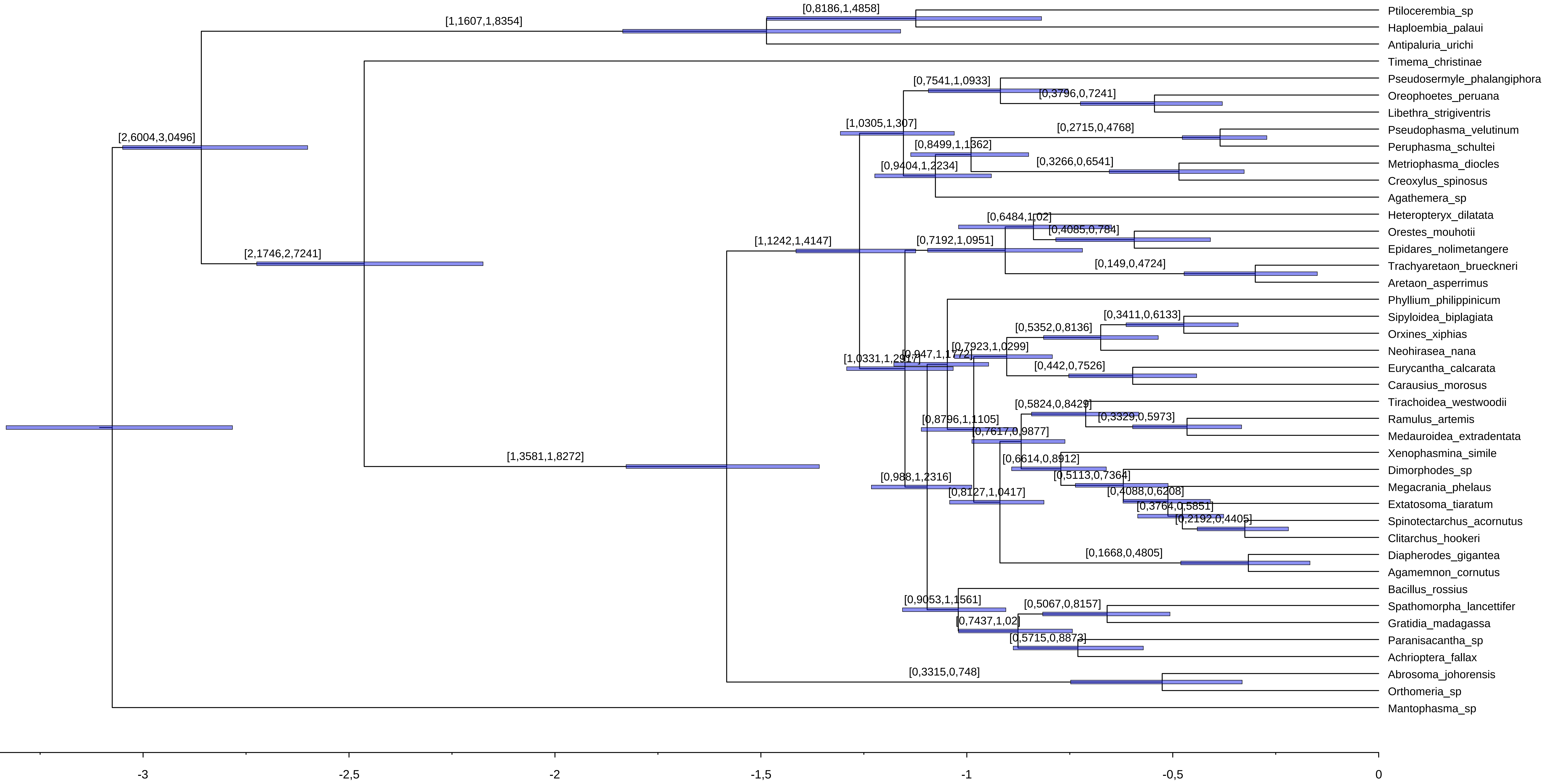

Ptilocerembia_sp
Haploembia_palaui
Antipaluria_urichi
Timema_christinae
Pseudosermyle_phalangiphora
Oreophoetes_peruana
Libethra_strigiventris
Pseudophasma_velutinum
Peruphasma_schultei
Metriophasma_diocles
Creoxylus_spinosus
Agathemera_sp
Heteropteryx_dilatata
Orestes_mouhotii
Epidares_nolimetangere
Trachyaretaon_brueckneri
Aretaon_asperrimus
Phyllium_philippinicum
Sipyloidea_biplagiata
Orxines_xiphias
Neohirasea_nana
Eurycantha_calcarata
Carausius_morosus
Tirachoidea_westwoodii
Ramulus_artemis
Medauroidea_extradentata
Xenophasmina_simile
Dimorphodes_sp
Megacrania_phelaus
Extatosoma_tiaratum
Spinotectarchus_acornutus
Clitarchus_hookeri
Diapherodes_gigantea
Agamemnon_cornutus
Bacillus_rossius
Spathomorpha_lancettifer
Gratidia_madagassa
Paranisacantha_sp
Achrioptera_fallax
Abrosoma_johorensis
Orthomeria_sp
Mantophasma_sp
[0,8186,1,4858]
[1,1607,1,8354]
[2,6004,3,0496]
[2,1746,2,7241]
[1,3581,1,8272]
[1,1242,1,4147]
[0,7541,1,0933]
[0,3796,0,7241]
[1,0305,1,307]
[0,2715,0,4768]
[0,8499,1,1362]
[0,3266,0,6541]
[0,9404,1,2234]
[0,6484,1,02]
[0,4085,0,784]
[0,7192,1,0951]
[0,149,0,4724]
[0,3411,0,6133]
[0,5352,0,8136]
[0,7923,1,0299]
[0,947,1,1772]
[1,0331,1,2917]
[0,442,0,7526]
[0,5824,0,8429]
[0,3329,0,5973]
[0,8796,1,1105]
[0,7617,0,9877]
[0,6614,0,8912]
[0,5113,0,7364]
[0,988,1,2316]
[0,4088,0,6208]
[0,8127,1,0417]
[0,3764,0,5851]
[0,2192,0,4405]
[0,1668,0,4805]
[0,9053,1,1561]
[0,5067,0,8157]
[0,7437,1,02]
[0,5715,0,8873]
[0,3315,0,748]
-3
-2,5
-2
-1,5
-1
-0,5
0

Figure S6

Dated phylogenetic tree of Phasmatodea based on the PhyloBayes re-analysis of the transcriptome dataset of Simon et al. (2019), excluding distantly related outgroups and using five fossil calibrations, i.e. without *Echinosomiscus primoticus*. Uniform prior distribution, autocorrelated rate clock.

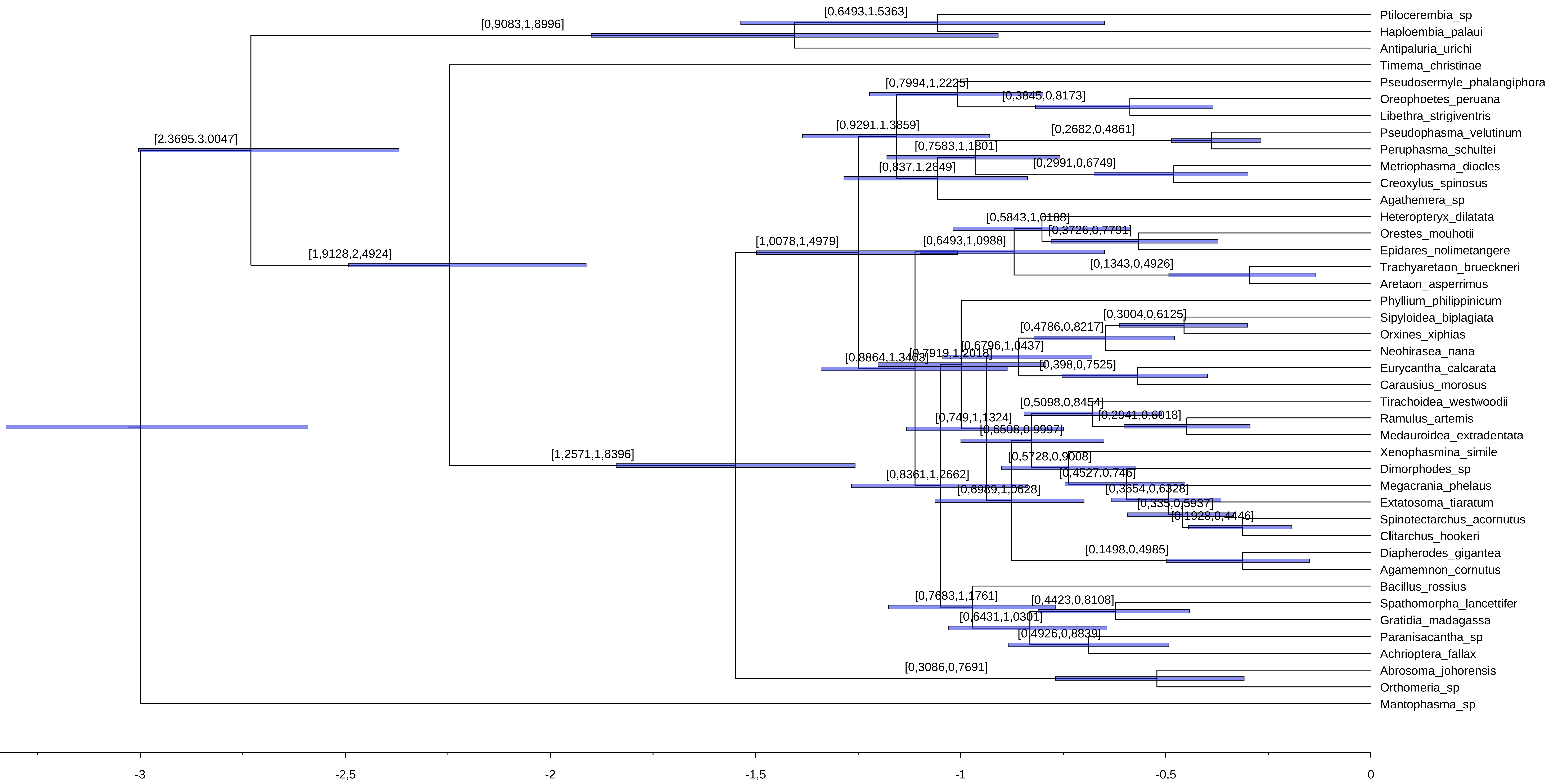

Ptilocerembia_sp
Haploembia_palaui
Antipaluria_urichi
Timema_christinae
Pseudosermyle_phalangiphora
Oreophoetes_peruana
Libethra_strigiventris
Pseudophasma_velutinum
Peruphasma_schultei
Metriophasma_diocles
Creoxylus_spinosus
Agathemera_sp
Heteropteryx_dilatata
Orestes_mouhotii
Epidares_nolimetangere
Trachyaretaon_brueckneri
Aretaon_asperrimus
Phyllium_philippinicum
Sipyloidea_biplagiata
Orxines_xiphias
Neohirasea_nana
Eurycantha_calcarata
Carausius_morosus
Tirachoidea_westwoodii
Ramulus_artemis
Medauroidea_extradentata
Xenophasmina_simile
Dimorphodes_sp
Megacrania_phelaus
Extatosoma_tiaratum
Spinotectarchus_acornutus
Clitarchus_hookeri
Diapherodes_gigantea
Agamemnon_cornutus
Bacillus_rossius
Spathomorpha_lancettifer
Gratidia_madagassa
Paranisacantha_sp
Achrioptera_fallax
Abrosoma_johorensis
Orthomeria_sp
Mantophasma_sp
[0,6493,1,5363]
[0,9083,1,8996]
[0,7994,1,2225]
[0,3845,0,8173]
[0,9291,1,3859]
[0,2682,0,4861]
[0,7583,1,1801]
[0,2991,0,6749]
[0,837,1,2849]
[2,3695,3,0047]
[0,5843,1,0188]
[0,3726,0,7791]
[1,0078,1,4979]
[0,6493,1,0988]
[1,9128,2,4924]
[0,1343,0,4926]
[0,3004,0,6125]
[0,4786,0,8217]
[0,6796,1,0437]
[0,8864,1,3403]
[0,7919,1,2018]
[0,398,0,7525]
[0,5098,0,8454]
[0,2941,0,6018]
[0,749,1,1324]
[0,6508,0,9997]
[1,2571,1,8396]
[0,5728,0,9008]
[0,4527,0,746]
[0,8361,1,2662]
[0,6989,1,0628]
[0,3654,0,6828]
[0,335,0,5937]
[0,1928,0,4446]
[0,1498,0,4985]
[0,7683,1,1761]
[0,4423,0,8108]
[0,6431,1,0301]
[0,4926,0,8839]
[0,3086,0,7691]
-3
-2,5
-2
-1,5
-1
-0,5
0

Figure S7

Dated phylogenetic tree of Phasmatodea based on the PhyloBayes re-analysis of the transcriptome dataset of Simon et al. (2019), excluding distantly related outgroups and using six fossil calibrations. Uniform prior distribution, independent rate clocks. Cauchy 10%, autocorrelated rate clock.

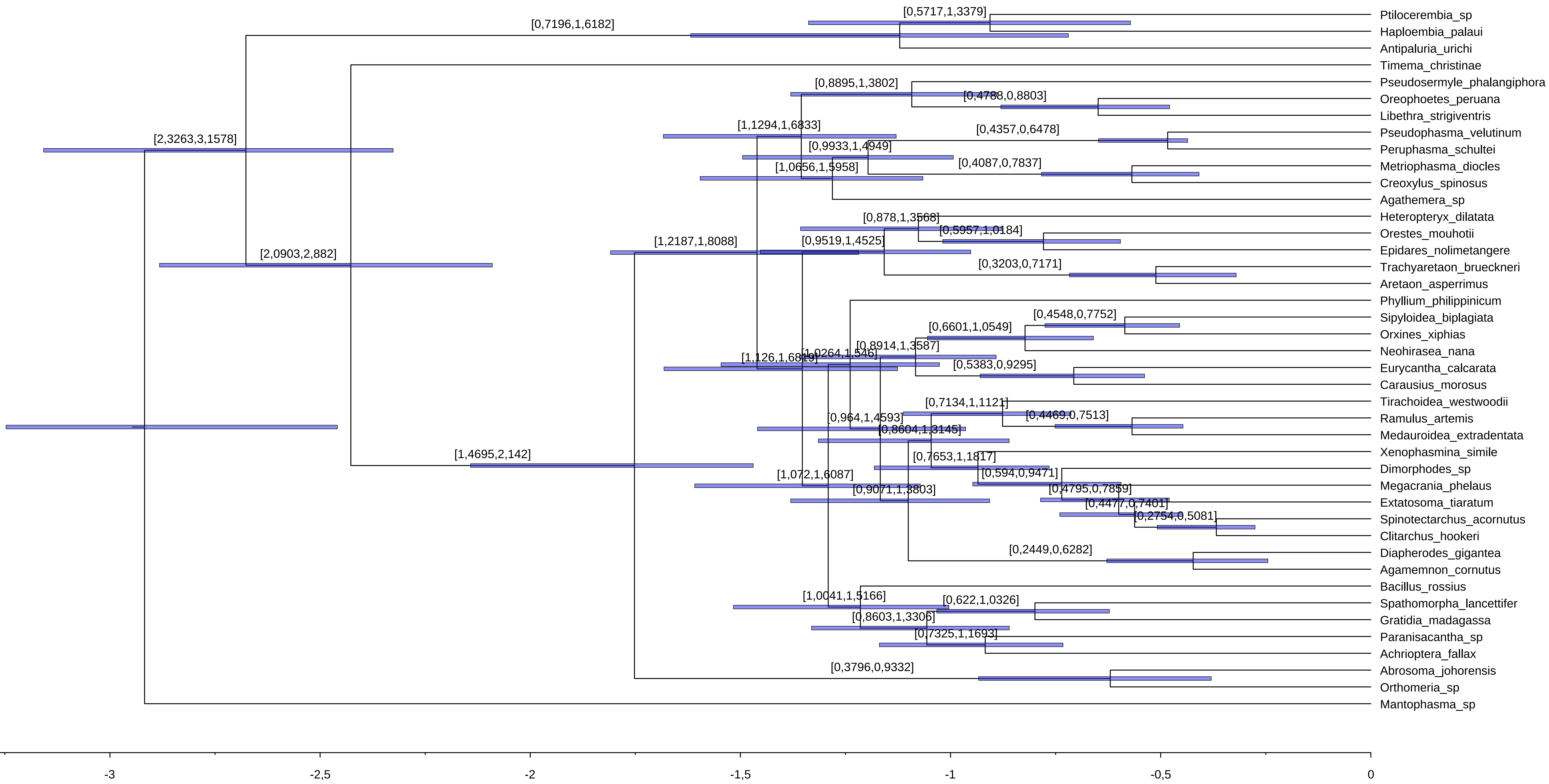
[0,5717,1,3379]
[0,7196,1,6182]
[2,3263,3,1578]
[0,8895,1,3802]
[0,4788,0,8803]
[1,1294,1,6833]
[0,4357,0,6478]
[0,9933,1,4949]
[0,4087,0,7837]
[1,0656,1,5958]
[0,878,1,3568]
[0,5957,1,0184]
[1,2187,1,8088]
[0,9519,1,4525]
[2,0903,2,882]
[0,3203,0,7171]
[0,4548,0,7752]
[0,6601,1,0549]
[0,8914,1,3587]
[1,126,1,6819]
[1,0264,1,546]
[0,5383,0,9295]
[0,7134,1,1121]
[0,4469,0,7513]
[0,964,1,4593]
[0,8604,1,3145]
[1,4695,2,142]
[0,7653,1,1817]
[0,594,0,9471]
[1,072,1,6087]
[0,9071,1,3803]
[0,4795,0,7859]
[0,4477,0,7401]
[0,2754,0,5081]
[0,2449,0,6282]
[1,0041,1,5166]
[0,622,1,0326]
[0,8603,1,3306]
[0,7325,1,1693]
[0,3796,0,9332]
Ptilocerembia_sp
Haploembia_palaui
Antipaluria_urichi
Timema_christinae
Pseudosermyle_phalangiphora
Oreophoetes_peruana
Libethra_strigiventris
Pseudophasma_velutinum
Peruphasma_schultei
Metriophasma_diocles
Creoxylus_spinosus
Agathemera_sp
Heteropteryx_dilatata
Orestes_mouhotii
Epidares_nolimetangere
Trachyaretaon_brueckneri
Aretaon_asperrimus
Phyllium_philippinicum
Sipyloidea_biplagiata
Orxines_xiphias
Neohirasea_nana
Eurycantha_calcarata
Carausius_morosus
Tirachoidea_westwoodii
Ramulus_artemis
Medauroidea_extradentata
Xenophasmina_simile
Dimorphodes_sp
Megacrania_phelaus
Extatosoma_tiaratum
Spinotectarchus_acornutus
Clitarchus_hookeri
Diapherodes_gigantea
Agamemnon_cornutus
Bacillus_rossius
Spathomorpha_lancettifer
Gratidia_madagassa
Paranisacantha_sp
Achrioptera_fallax
Abrosoma_johorensis
Orthomeria_sp
Mantophasma_sp
-3
-2,5
-2
-1,5
-1
-0,5
0

Figure S8

Dated phylogenetic tree of Phasmatodea based on the PhyloBayes re-analysis of the transcriptome dataset of Simon et al. (2019), excluding distantly related outgroups and using six fossil calibrations. Uniform prior distribution, independent rate clocks. Cauchy 10%, independent rate clock.

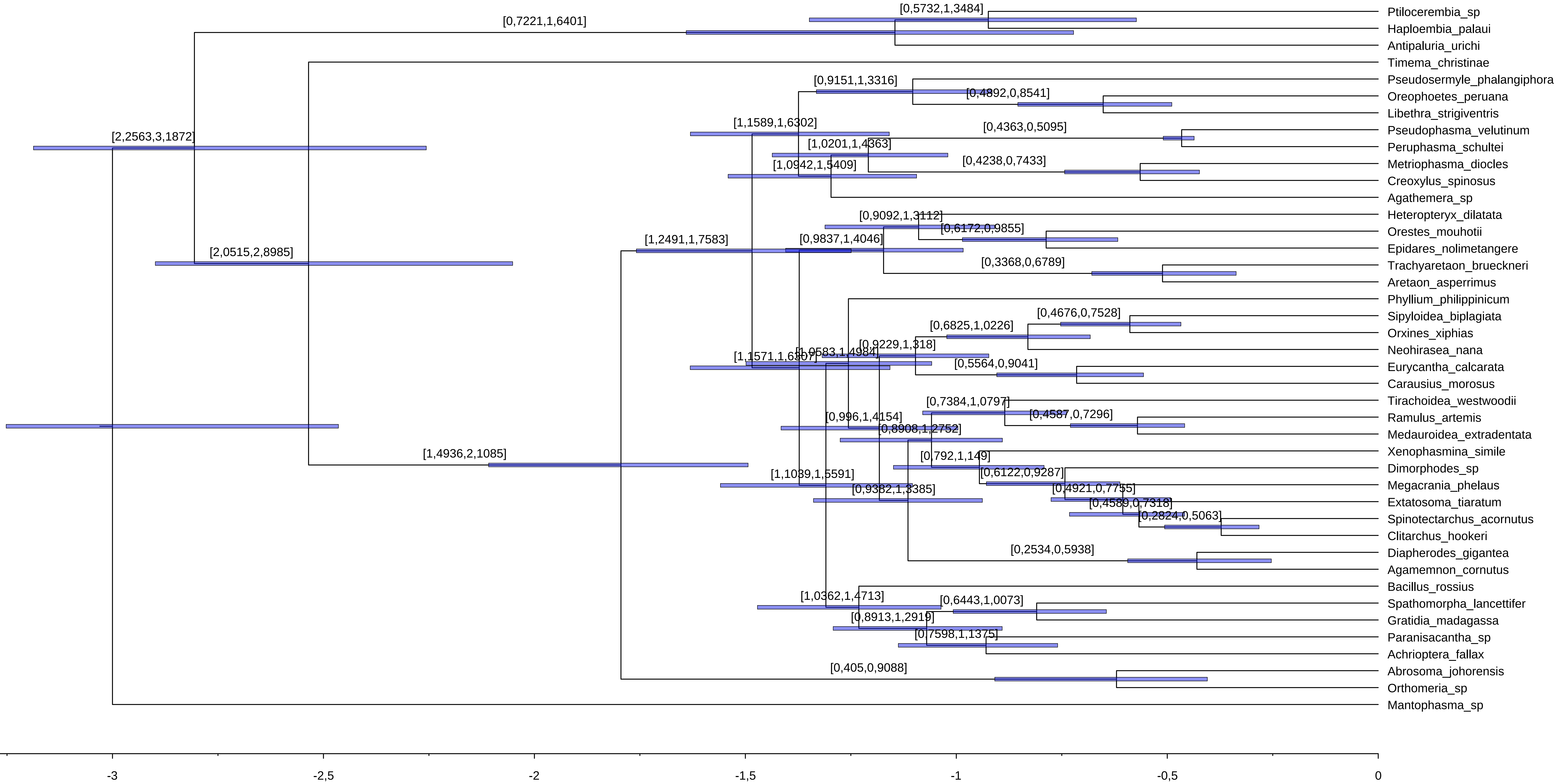

Ptilocerembia_sp
Haploembia_palaui
Antipaluria_urichi
Timema_christinae
Pseudosermyle_phalangiphora
Oreophoetes_peruana
Libethra_strigiventris
Pseudophasma_velutinum
Peruphasma_schultei
Metriophasma_diocles
Creoxylus_spinosus
Agathemera_sp
Heteropteryx_dilatata
Orestes_mouhotii
Epidares_nolimetangere
Trachyaretaon_brueckneri
Aretaon_asperrimus
Phyllium_philippinicum
Sipyloidea_biplagiata
Orxines_xiphias
Neohirasea_nana
Eurycantha_calcarata
Carausius_morosus
Tirachoidea_westwoodii
Ramulus_artemis
Medauroidea_extradentata
Xenophasmina_simile
Dimorphodes_sp
Megacrania_phelaus
Extatosoma_tiaratum
Spinotectarchus_acornutus
Clitarchus_hookeri
Diapherodes_gigantea
Agamemnon_cornutus
Bacillus_rossius
Spathomorpha_lancettifer
Gratidia_madagassa
Paranisacantha_sp
Achrioptera_fallax
Abrosoma_johorensis
Orthomeria_sp
Mantophasma_sp
[0,5732,1,3484]
[0,7221,1,6401]
[0,9151,1,3316]
[0,4892,0,8541]
[2,2563,3,1872]
[1,1589,1,6302]
[0,4363,0,5095]
[1,0201,1,4363]
[0,4238,0,7433]
[1,0942,1,5409]
[0,9092,1,3112]
[0,6172,0,9855]
[1,2491,1,7583]
[0,9837,1,4046]
[2,0515,2,8985]
[0,3368,0,6789]
[0,4676,0,7528]
[0,6825,1,0226]
[0,9229,1,318]
[1,0583,1,4984]
[1,1571,1,6507]
[0,5564,0,9041]
[0,7384,1,0797]
[0,996,1,4154]
[0,4587,0,7296]
[0,8908,1,2752]
[1,4936,2,1085]
[0,792,1,149]
[1,1039,1,5591]
[0,6122,0,9287]
[0,9382,1,3385]
[0,4921,0,7755]
[0,4589,0,7318]
[0,2824,0,5063]
[0,2534,0,5938]
[1,0362,1,4713]
[0,6443,1,0073]
[0,8913,1,2919]
[0,7598,1,1375]
[0,405,0,9088]
-3
-2,5
-2
-1,5
-1
-0,5
0

Figure S9

Dated phylogenetic tree of Phasmatodea based on the PhyloBayes re-analysis of the transcriptome dataset of Simon et al. (2019), excluding distantly related outgroups and using six fossil calibrations. Uniform prior distribution, independent rate clocks. Cauchy 50%, autocorrelated rate clock.

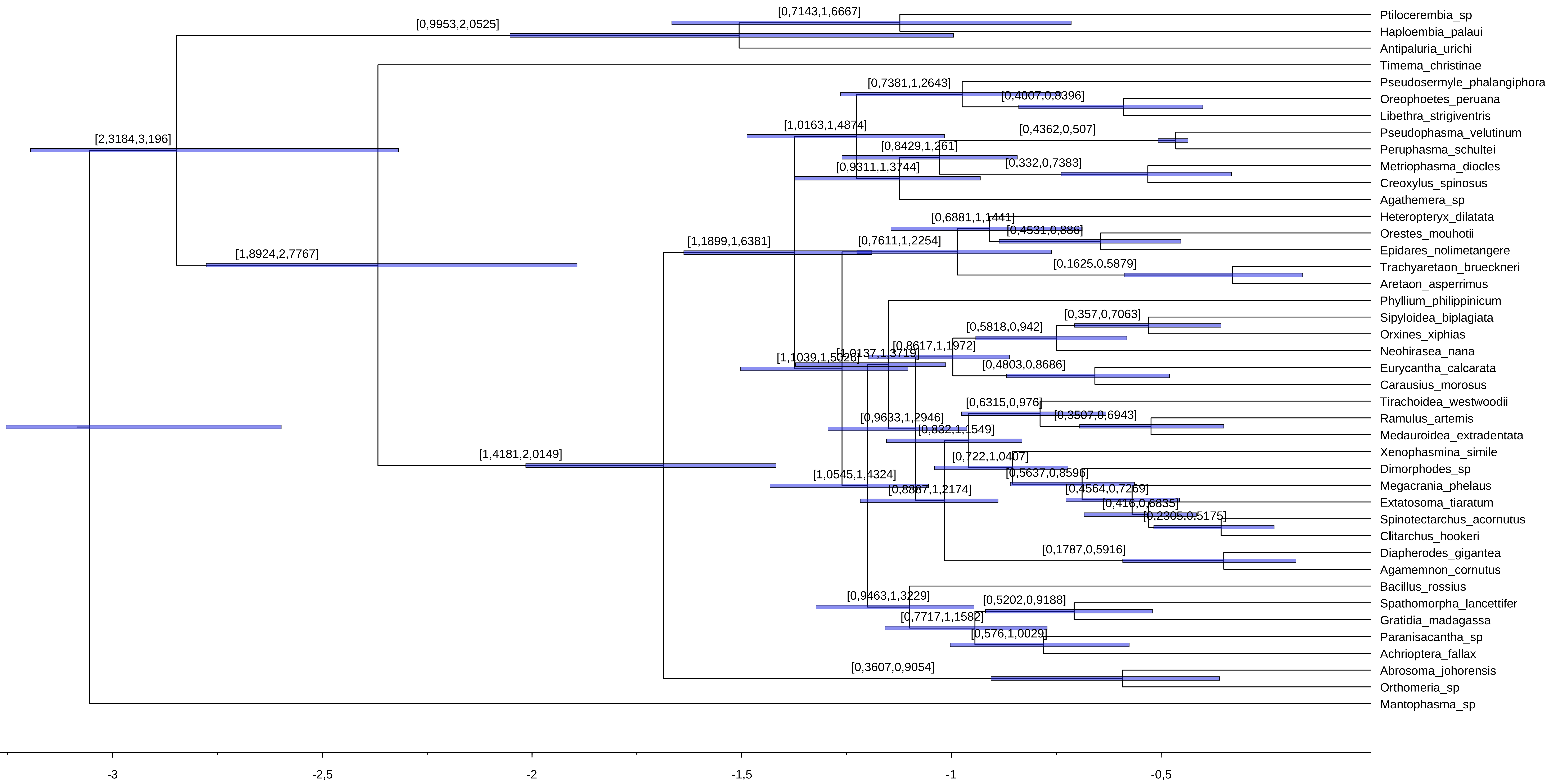
Ptilocerembia_sp
Haploembia_palaui
Antipaluria_urichi
Timema_christinae
Pseudosermyle_phalangiphora
Oreophoetes_peruana
Libethra_strigiventris
Pseudophasma_velutinum
Peruphasma_schultei
Metriophasma_diocles
Creoxylus_spinosus
Agathemera_sp
Heteropteryx_dilatata
Orestes_mouhotii
Epidares_nolimetangere
Trachyaretaon_brueckneri
Aretaon_asperrimus
Phyllium_philippinicum
Sipyloidea_biplagiata
Orxines_xiphias
Neohirasea_nana
Eurycantha_calcarata
Carausius_morosus
Tirachoidea_westwoodii
Ramulus_artemis
Medauroidea_extradentata
Xenophasmina_simile
Dimorphodes_sp
Megacrania_phelaus
Extatosoma_tiaratum
Spinotectarchus_acornutus
Clitarchus_hookeri
Diapherodes_gigantea
Agamemnon_cornutus
Bacillus_rossius
Spathomorpha_lancettifer
Gratidia_madagassa
Paranisacantha_sp
Achrioptera_fallax
Abrosoma_johorensis
Orthomeria_sp
Mantophasma_sp
[0,7143,1,6667]
[0,9953,2,0525]
[2,3184,3,196]
[1,8924,2,7767]
[0,7381,1,2643]
[0,4007,0,8396]
[1,0163,1,4874]
[0,4362,0,507]
[0,8429,1,261]
[0,9311,1,3744]
[0,332,0,7383]
[0,6881,1,1441]
[0,4531,0,886]
[1,1899,1,6381]
[0,7611,1,2254]
[0,1625,0,5879]
[0,357,0,7063]
[0,5818,0,942]
[0,8617,1,1972]
[1,0137,1,3719]
[1,1039,1,5026]
[0,4803,0,8686]
[0,6315,0,976]
[0,3507,0,6943]
[0,9633,1,2946]
[0,832,1,1549]
[1,4181,2,0149]
[0,722,1,0407]
[0,5637,0,8596]
[1,0545,1,4324]
[0,8887,1,2174]
[0,4564,0,7269]
[0,416,0,6835]
[0,2305,0,5175]
[0,1787,0,5916]
[0,9463,1,3229]
[0,5202,0,9188]
[0,7717,1,1582]
[0,576,1,0029]
[0,3607,0,9054]
-3
-2,5
-2
-1,5
-1
-0,5

Figure S10

Dated phylogenetic tree of Phasmatodea based on the PhyloBayes re-analysis of the transcriptome dataset of Simon et al. (2019), excluding distantly related outgroups and using six fossil calibrations. Uniform prior distribution, independent rate clocks. Cauchy 50%, independent rate clock.

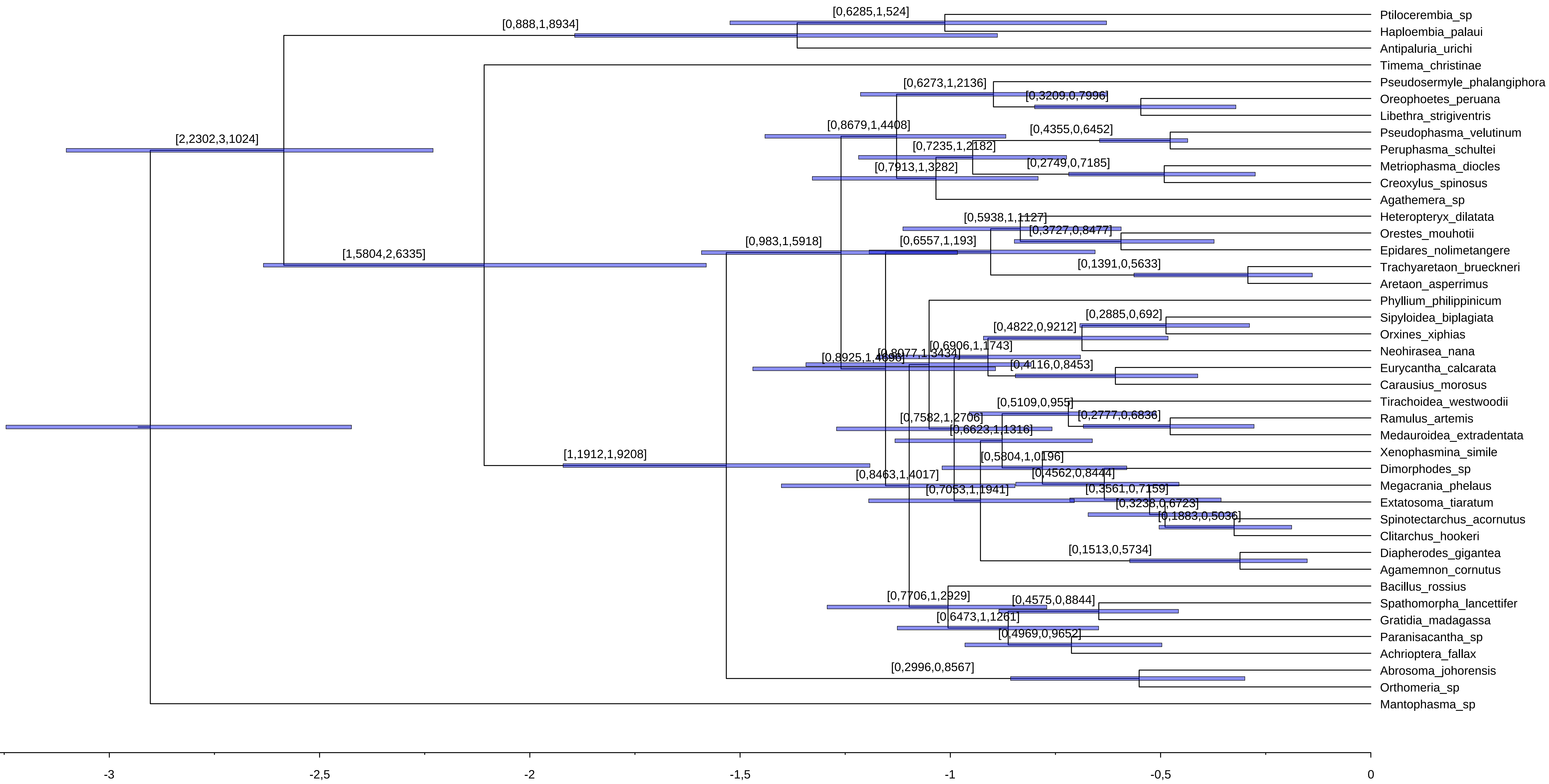

[0,6285,1,524]
[0,888,1,8934]
[2,2302,3,1024]
[0,6273,1,2136]
[0,3209,0,7996]
[0,8679,1,4408]
[0,4355,0,6452]
[0,7235,1,2182]
[0,7913,1,3282]
[0,2749,0,7185]
[0,5938,1,1127]
[0,3727,0,8477]
[1,5804,2,6335]
[0,983,1,5918]
[0,6557,1,193]
[0,1391,0,5633]
[0,2885,0,692]
[0,4822,0,9212]
[0,6906,1,1743]
[0,8077,1,3434]
[0,8925,1,4696]
[0,4116,0,8453]
[0,5109,0,955]
[0,2777,0,6836]
[0,7582,1,2706]
[0,6623,1,1316]
[1,1912,1,9208]
[0,5804,1,0196]
[0,4562,0,8444]
[0,8463,1,4017]
[0,7053,1,1941]
[0,3561,0,7159]
[0,3238,0,6723]
[0,1883,0,5036]
[0,1513,0,5734]
[0,7706,1,2929]
[0,4575,0,8844]
[0,6473,1,1261]
[0,4969,0,9652]
[0,2996,0,8567]
Ptilocerembia_sp
Haploembia_palaui
Antipaluria_urichi
Timema_christinae
Pseudosermyle_phalangiphora
Oreophoetes_peruana
Libethra_strigiventris
Pseudophasma_velutinum
Peruphasma_schultei
Metriophasma_diocles
Creoxylus_spinosus
Agathemera_sp
Heteropteryx_dilatata
Orestes_mouhotii
Epidares_nolimetangere
Trachyaretaon_brueckneri
Aretaon_asperrimus
Phyllium_philippinicum
Sipyloidea_biplagiata
Orxines_xiphias
Neohirasea_nana
Eurycantha_calcarata
Carausius_morosus
Tirachoidea_westwoodii
Ramulus_artemis
Medauroidea_extradentata
Xenophasmina_simile
Dimorphodes_sp
Megacrania_phelaus
Extatosoma_tiaratum
Spinotectarchus_acornutus
Clitarchus_hookeri
Diapherodes_gigantea
Agamemnon_cornutus
Bacillus_rossius
Spathomorpha_lancettifer
Gratidia_madagassa
Paranisacantha_sp
Achrioptera_fallax
Abrosoma_johorensis
Orthomeria_sp
Mantophasma_sp
-3
-2,5
-2
-1,5
-1
-0,5
0

Figure S11

Dated phylogenetic tree of Phasmatodea based on the PhyloBayes re-analysis of the transcriptome dataset of Simon et al. (2019), excluding distantly related outgroups and using six fossil calibrations. Uniform prior distribution, independent rate clocks. Cauchy 90%, autocorrelated rate clock.

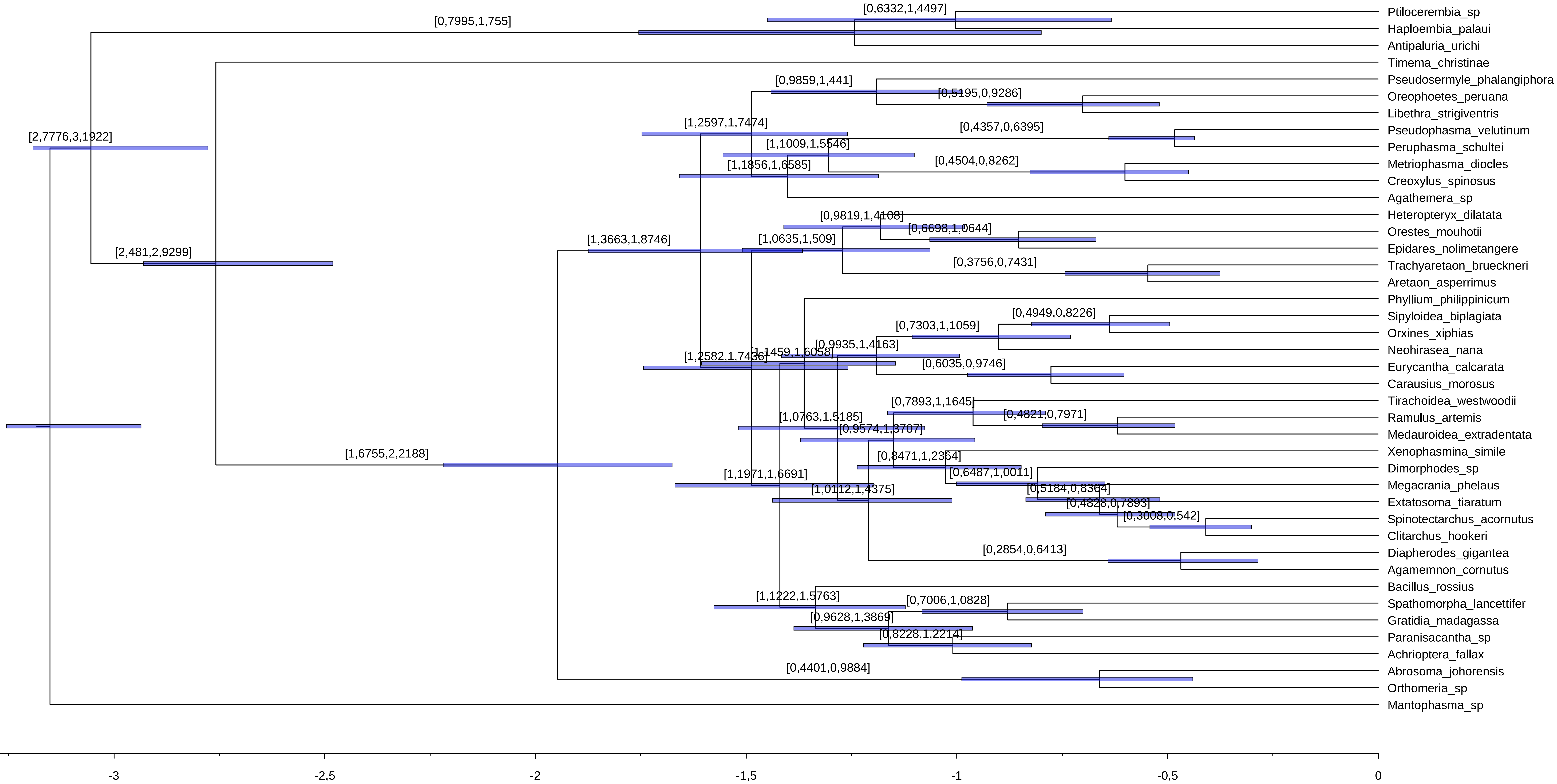

Ptilocerembia_sp
Haploembia_palaui
Antipaluria_urichi
Timema_christinae
Pseudosermyle_phalangiphora
Oreophoetes_peruana
Libethra_strigiventris
Pseudophasma_velutinum
Peruphasma_schultei
Metriophasma_diocles
Creoxylus_spinosus
Agathemera_sp
Heteropteryx_dilatata
Orestes_mouhotii
Epidares_nolimetangere
Trachyaretaon_brueckneri
Aretaon_asperrimus
Phyllium_philippinicum
Sipyloidea_biplagiata
Orxines_xiphias
Neohirasea_nana
Eurycantha_calcarata
Carausius_morosus
Tirachoidea_westwoodii
Ramulus_artemis
Medauroidea_extradentata
Xenophasmina_simile
Dimorphodes_sp
Megacrania_phelaus
Extatosoma_tiaratum
Spinotectarchus_acornutus
Clitarchus_hookeri
Diapherodes_gigantea
Agamemnon_cornutus
Bacillus_rossius
Spathomorpha_lancettifer
Gratidia_madagassa
Paranisacantha_sp
Achrioptera_fallax
Abrosoma_johorensis
Orthomeria_sp
Mantophasma_sp
[0,6332,1,4497]
[0,7995,1,755]
[0,9859,1,441]
[0,5195,0,9286]
[2,7776,3,1922]
[1,2597,1,7474]
[0,4357,0,6395]
[1,1009,1,5546]
[0,4504,0,8262]
[1,1856,1,6585]
[0,9819,1,4108]
[0,6698,1,0644]
[1,3663,1,8746]
[1,0635,1,509]
[2,481,2,9299]
[0,3756,0,7431]
[0,4949,0,8226]
[0,7303,1,1059]
[0,9935,1,4163]
[1,1459,1,6058]
[1,2582,1,7436]
[0,6035,0,9746]
[0,7893,1,1645]
[0,4821,0,7971]
[1,0763,1,5185]
[0,9574,1,3707]
[1,6755,2,2188]
[0,8471,1,2364]
[0,6487,1,0011]
[1,1971,1,6691]
[1,0112,1,4375]
[0,5184,0,8364]
[0,4828,0,7893]
[0,3008,0,542]
[0,2854,0,6413]
[1,1222,1,5763]
[0,7006,1,0828]
[0,9628,1,3869]
[0,8228,1,2214]
[0,4401,0,9884]
-3
-2,5
-2
-1,5
-1
-0,5
0

Figure S12

Dated phylogenetic tree of Phasmatodea based on the PhyloBayes re-analysis of the transcriptome dataset of Simon et al. (2019), excluding distantly related outgroups and using six fossil calibrations. Uniform prior distribution, independent rate clocks. Cauchy 90%, independent rate clock.

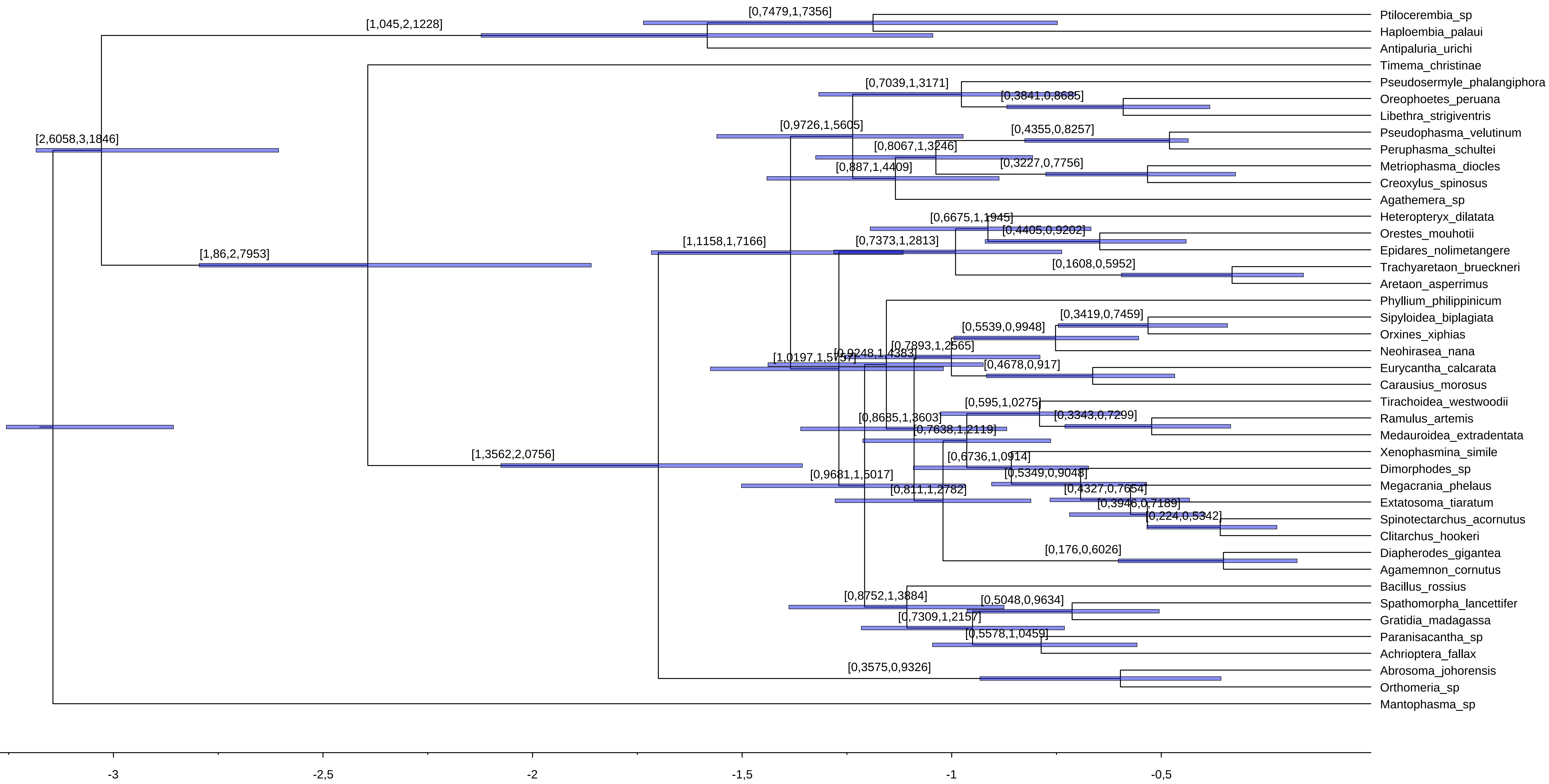

Ptilocerembia_sp
Haploembia_palaui
Antipaluria_urichi
Timema_christinae
Pseudosermyle_phalangiphora
Oreophoetes_peruana
Libethra_strigiventris
Pseudophasma_velutinum
Peruphasma_schultei
Metriophasma_diocles
Creoxylus_spinosus
Agathemera_sp
Heteropteryx_dilatata
Orestes_mouhotii
Epidares_nolimetangere
Trachyaretaon_brueckneri
Aretaon_asperrimus
Phyllium_philippinicum
Sipyloidea_biplagiata
Orxines_xiphias
Neohirasea_nana
Eurycantha_calcarata
Carausius_morosus
Tirachoidea_westwoodii
Ramulus_artemis
Medauroidea_extradentata
Xenophasmina_simile
Dimorphodes_sp
Megacrania_phelaus
Extatosoma_tiaratum
Spinotectarchus_acornutus
Clitarchus_hookeri
Diapherodes_gigantea
Agamemnon_cornutus
Bacillus_rossius
Spathomorpha_lancettifer
Gratidia_madagassa
Paranisacantha_sp
Achrioptera_fallax
Abrosoma_johorensis
Orthomeria_sp
Mantophasma_sp
[0,7479,1,7356]
[1,045,2,1228]
[2,6058,3,1846]
[0,7039,1,3171]
[0,3841,0,8685]
[0,9726,1,5605]
[0,4355,0,8257]
[0,8067,1,3246]
[0,887,1,4409]
[0,3227,0,7756]
[0,6675,1,1945]
[0,4405,0,9202]
[1,1158,1,7166]
[0,7373,1,2813]
[1,86,2,7953]
[0,1608,0,5952]
[0,3419,0,7459]
[0,5539,0,9948]
[0,7893,1,2565]
[0,9248,1,4383]
[1,0197,1,5757]
[0,4678,0,917]
[0,595,1,0275]
[0,3343,0,7299]
[0,8685,1,3603]
[0,7638,1,2119]
[1,3562,2,0756]
[0,6736,1,0914]
[0,9681,1,5017]
[0,5349,0,9048]
[0,811,1,2782]
[0,4327,0,7654]
[0,3946,0,7189]
[0,224,0,5342]
[0,176,0,6026]
[0,8752,1,3884]
[0,5048,0,9634]
[0,7309,1,2157]
[0,5578,1,0459]
[0,3575,0,9326]
-3
-2,5
-2
-1,5
-1
-0,5

Supplementary references

Archibald SB, Bradler S. Stem-group stick insects (Phasmatodea) in the early Eocene at McAbee, British Columbia, Canada, and Republic, Washington, United States of America. Can Entomol. 2015;147:744–753.

Bestland EA, Hammond PE, Blackwell DLS, Kays MA, Retallack GJ, Stimac J. Geologic framework of the Clarno Unit, John Day Fossil Beds National Monument, Central Oregon. Oregon Geol. 1999;61:3–19.

Bradler S. The vomer of *Timema* Scudder, 1895 (Insecta: Phasmatodea) and its significance for phasmatodean phylogeny. Cour Forsch-Inst Senckenberg. 1999;215:43–47.

Bradler S. Die Phylogenie der Stab- und Gespentschrecken (Insecta: Phasmatodea). Spec Phyl Evol. 2009;2:3–139.

Bradler S. Der phasmatodea tree of life: überraschendes und ungeklärtes in der stabschrecken-evolution. Entomol. Heut. 2015;27:1–23.

Beier M. Orthopteroidea. Ordnung: Cheleutoptera Crampton 1915 (Phasmida Leach 1815). In: Weber H, editor. Dr. H. G. Bronns Klassen und Ordnungen des Tierreichs. V. Arthropoda, III. Abteilung: Insecta. Leipzig: Akademische Verlagsgesellschaft; 1957. p. 305–454.

Bradler S, Cliquennois N, Buckley TR. Single origin of the Mascarene stick insects: ancient radiation on sunken islands? BMC Evol Biol. 2015;15:196.

Beier M. Phasmida (Stab- oder Gespenstheuschrecken). Handbuch der Zoologie IV. Berlin: Walter de Gruyter & Co; 1968.

Bradley JC, & Galil BS. The taxonomic arrangement of the Phasmatodea with keys to the subfamilies and tribes. Proc Entomol Soc Wash. 1977;79:176–208.

Brock PD, Hasenpusch JW. The Complete Field Guide to Stick and Leaf Insects of Australia. Clayton: CSIRO Publishing; 2009

Carpenter FM. Superclass Hexapoda. Treatise on Invertebrate Paleontology. Boulder, CO: The Geological Society of America and the University of Kansas, 1992.

Chen S, Yin X, Lin X, Shih C, Zhang R, Gao T, Ren D. Stick insect in Burmese amber reveals an early evolution of lateral lamellae in the Mesozoic. Proc Roy Soc B. 2018;285:20180425.

Chen S, Deng SW, Shih C, Zhang WW, Zhang P, Ren D, et al. The earliest Timematids in Burmese amber reveal diverse tarsal pads of stick insects in the mid-Cretaceous. Insect Sci. 2019;26:945–957.

Chen W, Ji Q, Liu DY, Zhang Y, Song B, Yu LX. Isotope geochronology of the fossil-bearing beds in the Daohugou area, Ningcheng, Inner Mongolia. Geol Bull Chin. 2004;23:1165–1169.

Clark-Sellick JT. Phasmida (stick insect) eggs from the Eocene of Oregon. Palaeontol. 1994;37: 913–922.

Clark-Sellick JT. Descriptive terminology of the phasmid egg capsule, with an extended key to the phasmid genera based on egg structure. Syst Entomol. 1997;22:97–122.

Cui Y, Shih C, Ren D. Notoptera–Rock Crawlers and Ice Crawlers. In: Ren D, Shih C, Gao T, Wang Y, Yao Y, editors. Rhythms of Insect Evolution: Evidence from the Jurassic and Cretaceous in Northern China. New York, NY: Willey Blackwell; 2019. p. 137–147.

Damgaard J, Klass KD, Picker MD, Buder G. Phylogeny of the Heelwalkers (Insecta: Mantophasmatodea) based on mtDNA sequences, with evidence for additional taxa in South Africa. Mol Phyl Evol. 2008;47,443–462.

Engel MS, Wang B, Alqarni AS. A thorny, 'anareolate' stick-insect (Phasmatidae sl) in Upper Cretaceous amber from Myanmar, with remarks on diversification times among Phasmatodea. Cret Res. 2016;63:45–53.

Friedemann K, Wipfler B, Bradler S, Beutel RG. On the head morphology of *Phyllium* and the phylogenetic relationships of Phasmatodea (Insecta). Acta Zool. 2012;93:184–199.

Gao K, Ren D. Radiometric dating of ignimbrite from Inner Mongolia Provides no indication of a Post-Middle Jurassic Age for the Daohugou Beds. Acta Geol Sin. 2006;80:42–45.

Grimaldi DA, Engel MS, Nascimbene PC. Fossiliferous Cretaceous amber from Myanmar (Burma): its rediscovery, biotic diversity, and paleontological significance. Am Mus Novit. 2002;3361:1–71.

Günther K. Über die taxonomische gliederung und geographische verbreitung der insektenordnung der Phasmatodea. Beit Entomol. 1953;3:541–563.

He HY,Wang XL, Zhou ZH, Zhu RX, Jin F, Wang F, Ding X, Boven A. 2004. 40Ar/ 39Ar dating of ignimbrite from Inner Mongolia, northeastern China, indicates a post-Middle Jurassic age for the overlying Daohugou Bed. Geophys Res Lett. 2004;31:L20609.

Heřmanová Z, Bodor E, Kvaček J. *Knoblochia cretacea*, Late Cretaceous insect eggs from Central Europe. Cret Res. 2013;45:7–15.

Huang, D. The Daohugou Biota. Shanghai: Shanghai Science and Technology; 2016.

Huang D, Nel A, Zompro O., Waller A. Mantophasmatodea now in the Jurassic. Naturwissenschaften. 2008;95:947–952.

Iturralde-Vinent MA, MacPhee RD. Age and paleogeographical origin of Dominican amber. Science. 1996;273:1850–1852.

Iturralde-Vinent MA, MacPhee RD. Remarks on the age of Dominican amber. Palaeoentomol. 2019;2,236–240.

Kevan DK. Phasmatoptera. In: Parker SF, editor. Synopsis and Classification of Living Organisms. Vol. 2. New York, NY: McGraw-Hill; 1982. p. 379–383.

Kohli MK, Ware JL, Bechly G. How to date a dragonfly: Fossil calibrations for odonates. Palaeontol Electr. 2016;19:1–14.

Kristensen NI. Phylogeny of hexapod "orders". A critical review of recent accounts. Z Zool Syst Evol. 1975;13:1–44.

Kryza R, Crowley QG, Larionov A, Pin C, Oberc-Dziedzic T, Mochnacka K. Chemical abrasion applied to SHRIMP zircon geochronology: an example from the Variscan Karkonosze Granite (Sudetes, SW Poland). Gondw Res. 2012;21:757–767.

Kukalová J. On the systematic position of the supposed Permian beetles, Tshecardocoleidae, with a description of a new collection from Moravia. Sb. geol. věd řada P. Paleontol. 1969;11:139–161.

Kukalová-Peck J, Tihelka E. The origin of insect wings: an interview with the Czech palaeoentomologist Jarmila Kukalová-Peck. Fossil News 2019;22:34–43.

Liu YQ, Liu YX, Li PX, Zhang H, Zhang LJ, et al. Daohugou biota-bearing lithostratigraphic succession on the southeastern margin of the Ningcheng basin, Inner Mongolia, and its geochronology. Geol Bull Chin. 2004;23:1180–1187.

Maksoud S, Azar D, Granier B, Gèze R. New data on the age of the Lower Cretaceous amber outcrops of Lebanon. Palaeoworld. 2017;26:331–338.

Manchester SR. Fruits and seeds of the middle Eocene Nut Beds flora, Clarno Formation, Oregon: Palaeontogr Am. 1994;58: 1–205.

Mao Y, Liang K, Su Y, Li J, Rao X, Zhang H, Xia F, et al . Various amberground marine animals on Burmese amber with discussions on its age. Palaeoentomol. 2018;1:91–93.

Metcalfe I, Crowley JL, Nicoll RS, Schmitz M. High-precision U-Pb CA-TIMS calibration of Middle Permian to Lower Triassic sequences, mass extinction and extreme climate-change in eastern Australian Gondwana. Gondw Res. 2015;28: 61–81.

Mihlbachler MC, Samuels JX. A small-bodied species of Brontotheriidae from the middle Eocene Nut Beds of the Clarno Formation, John Day Basin, Oregon. J Paleontol. 2016;90:1233–1244.

Misof B, Liu S, Meusemann K, Peters RS, Donath A, Mayer C, et al. Phylogenomics resolves the timing and pattern of insect evolution. Science. 2014;346:763–767.

Nel A, Delfosse E. A new Chinese Mesozoic stick insect. Acta Pal Pol. 2011;56: 429–432.

Ogg JG, Ogg GM, Gradstein FMA. Concise Geologic Time Scale Amsterdam: Elsevier; 2016.

Peng DC, Hong YC, Zhang ZJ. Namurian insects (Diaphanopterodea) from Qilianshan Mountains, China. Geol Not. 2005;24:219–234.

Poinar G. A walking stick, Clonistria dominicana n. sp. (Phasmatodea: Diapheromeridae) in Dominican amber. Hist Biol. 2011;23:223–6.

Poinar G. Burmese amber: evidence of Gondwanan origin and Cretaceous dispersion. Hist Biol. 2019;31:1304–1309 (2019).

Rasnitsyn AP, Ross AJ. A preliminary list of arthropod families present in the Burmese amber collection at the Natural History Museum, London. Bull Nat Hist Mus Geol Ser. 2000;56:21–24

Rasnitsyn AP. Quicke DLJ. History of Insects. Dordrecht: Kluwer Academic Publishers, 2002.

Shang L, Béthoux O, Ren D. New stem-Phasmatodea from the middle Jurassic of China. Eur J Entomol. 2011; 108: 677–685.

Sharov AG. Filogeniya orthopteroidnykh nasekomykh. Tr Paleontol Inst Akad Nauk SSSR. 1968;118:1–216.

Shcherbakov DE. Madygen, Triassic Lagerstätte number one, before and after Sharov. Alavesia. 2008;2:113–124.

Sherratt E, del Rosario Castañeda M, Garwood RJ, Mahler LD, Sanger TJ, Herrel A, de Queiroz K, Losos JB. Amber fossils demonstrate deep-time stability of Caribbean lizard communities. Proc Natl Acad Sci. 2015;112:9961–9966.

Shi G, Grimaldi DA, Harlow GE, Wang J, Wang J, Yang M, et al. Age constraint on Burmese amber based on U–Pb dating of zircons. Cret. Res. 2012;37:155–163.

Simon S, Letsch H, Bank S, Buckley T, Donath A, Liu S, et al. Old world and New world Phasmatodea: phylogenomics resolve the evolutionary history of stick and leaf insects. Front Ecol Evol. 2019;7:345.

Swisher CC, 40Ar/39Ar dating and its application to the calibration of the North American Land Mammal ages. Ph.D. dissertation, Berkeley: University of California; 1992.

Terry MD, Whiting MF. Mantophasmatodea and phylogeny of the lower neopterous insects. Cladistics. 2005;21:240-257.

Tilgner E. The fossil record of Phasmida (Insecta: Neoptera). Insect Syst Evol. 2000;31,473–480.

Tilgner EH, Kiselyova TG, McHugh JV. A morphological study of *Timema cristinae* Vickery with implications for the phylogenetics of Phasmida. D Entomol Z. 1999;46:149–162.

Wang M, Béthoux O, Bradler S, Jacques FM, Cui Y, Ren D. Under cover at pre-angiosperm times: a cloaked phasmatodean insect from the Early Cretaceous Jehol biota. PloS One. 2014;9: e91290.

Wang X, Zhou Z, He H, Fan J, Wang Y, Zhang J, Wang Y, Zhang F. Stratigraphy and age of the Daohugou Bed in Ningcheng, Inner Mongolia. Chin Sci Bull. 2005;50:2369.

Wheeler WC, Giribet G, Edgecombe GD. Arthropod systematics: The comparative study of genomic, anatomical and paleontological information. In: Cracraft J, Donoghue MJ, editors. Assembling the Tree of Life. Oxford, Oxford University Press, 2004.

Whiting MF, Bradler S, Maxwell T. Loss and recovery of wings in stick insects. Nature. 2003;421:264–267.

Willmann R. Die phylogenetischen beziehungen der Insecta: offene fragen und probleme. Verh. Westdeut Entomol. 2003;2001:1–64.

Yang H, Yin X, Lin X, Wang C, Shih C, Zhang W, et al. Cretaceous winged stick insects clarify the early evolution of Phasmatodea. Proc Roy Soc B. 2019;286: 20191085.

Yang H, Shi C, Engel MS, Zhao Z, Ren D, Gao T. Early specializations for mimicry and defense in a Jurassic stick insect. Natl Sci Rev. 2020;287:nwaa056, in press.

Yu T, Kelly R, Mu L, Ross A, Kennedy J, Broly P, et al. An ammonite trapped in Burmese amber. Proc Natl Acad Sci. 2019;116:11345–11350.

Zhang J. Archisargoid flies (Diptera, Brachycera, Archisargidae and Kovalevisargidae) from the Jurassic Daohugou biota of China, and the related biostratigraphical correlation and geological age. J Syst Palaentol. 2015;13:857–881.

Zhang Z, Schneider JW., Hong, Y. The most ancient roach (Blattodea): a new genus and species from the earliest Late Carboniferous (Namurian) of China, with a discussion of the phylomorphogeny of early blattids. J Syst Palaeontol. 2013;11:27–40.

Zompro O. Inter- and intra-ordinal relationships of the Mantophasmatodea, with comments on the phylogeny of polyneopteran orders (Insecta: Polyneoptera). Mitt Geol-Pal Inst Univ Hamburg. 2005;89:85–116.

Zompro O. *Raptophasma groehni* n. sp., a new species of gladiator from Baltic amber (Insecta: Mantophasmatodea: Mantophasmatidae). Arthropoda. 2008;16:26–27.

Zompro O, Adis J, Weitschat W. A review of the order Mantophasmatodea (Insecta). Zool Anz. 2002;241:269–279.
